# The cost-effectiveness of treating chronic hepatitis B patients in a median endemic and middle income country

**DOI:** 10.1007/s10198-012-0413-8

**Published:** 2012-07-20

**Authors:** Mehlika Toy, Fatih Oguz Onder, Ramazan Idilman, Gokhan Kabacam, Jan Hendrik Richardus, Mithat Bozdayi, Meral Akdogan, Zarife Kuloglu, Aydan Kansu, Solko Schalm, Cihan Yurdaydin

**Affiliations:** 1 Department of Public Health, Erasmus MC, University Medical Center Rotterdam, Dr. Molewaterplein 50, 3000 CA Rotterdam, The Netherlands; 2 LiverDoc, Rotterdam, The Netherlands; 3 Department of Gastroenterology, Yuksek Ihtisas Hospital, Ankara, Turkey; 4 Department of Gastroenterology, School of Medicine, Ankara University, Ankara, Turkey; 5 Hepatology Institute, School of Medicine, Ankara, Turkey; 6 Department of Pediatric Gastroenterology and Hepatology, School of Medicine, Ankara, Turkey; 7 Department of Gastroenterology and Hepatology, Erasmus MC, University Medical Center Rotterdam, Rotterdam, The Netherlands

**Keywords:** Chronic hepatitis B, Cost-effectiveness analysis, Antiviral therapy, Turkey, Middle income, I18

## Abstract

**Background/aims:**

Chronic hepatitis B (CHB) infection is a serious public health problem due to its potential liver disease sequelae and highly expensive medical costs such as the need for liver transplantation. The aim of this study was to quantify the burden of active CHB in terms of mortality and morbidity, the eligibility of antiviral treatment and to assess various treatment scenarios and possible salvage combinations for cost-effectiveness.

**Methods:**

A population cohort from a large data base of chronic hepatitis B patients was constructed and stratified according to 10-year age groups, the prevalence of HBsAg, HBV DNA level, ALT level, HBeAg status and the presence of cirrhosis. An age-specific Markov model for disease progression and cost-effectiveness analysis was constructed and calibrated for the specific population setting.

**Results:**

Of about 3.2 million estimated HBsAg carriers, 25 % are eligible for treatment. If the active cohort remains untreated, 31 % will die due to liver related complications. Within a 20-year period, 11 % will have developed decompensated cirrhosis, 12 % liver cancer and 6 % will need liver transplantation. Quality adjusted life years (QALYs) for the no treatment scenario ranged from 9.3 to 14.0. For scenarios with antiviral treatment, QALYs ranged from 9.9 to 14.5 for lamivudine, 13.0–17.5 for salvage therapy, and 16.6–19.0 for the third generation drugs entecavir and tenofovir.

**Conclusion:**

In a country with considerable amount of active CHB patients, monotherapy with a highly potent third generation drug has the most health-gain, and is cost-effective in both HBeAg-positive and negative in all stages of liver disease.

**Electronic supplementary material:**

The online version of this article (doi:10.1007/s10198-012-0413-8) contains supplementary material, which is available to authorized users.

## Introduction

Chronic hepatitis B (CHB) is a major global public health problem and an important cause of morbidity and mortality from sequelae related to CHB which includes cirrhosis development, decompensation and hepatocellular carcinoma [1].

Antiviral therapy is the only option to control and prevent progression of disease in chronic patients. The indications are generally the same for HBeAg-positive and negative patients. These are based mainly on the combination of three criteria: serum HBV DNA and ALT levels, and the stage of liver disease [2].

The course from infection exposure to the development of complications related to CHB infection may span multiple decades. Once diagnosed, treatment may modify the natural course for the better. The American and European guidelines on treatment of chronic hepatitis B recommend treatment with pegylated interferon or the nucleos(t)ide analogs (NA) entecavir or tenofovir [2, 3]. The latter two NAs are preferred over other NAs because of their antiviral potency and a high genetic barrier to resistance. However, treatment options need to be balanced in resource constraint settings. It should be of global concern that resource limitations are especially evident where hepatitis B is endemic or hyperendemic such as in the Far East or in Sub-Saharan Africa [4, 5]. The consequences and costs of treatment strategies may help in contributing to the buildup of health strategies. Based on its GNI (gross national income) per capita, every economy is classified as low income, middle income (subdivided into lower middle and upper middle), or high income, according to the World Bank. The GNI per capita for Turkey is $ 9,500, which classifies Turkey as an upper middle income country. To support national health authorities policy making in long-term chronic hepatitis B treatment, we assessed the impact of treatment in preventing adverse outcomes of CHB infection, and the cost-effectiveness of various treatment strategies. For these goals, Turkey was used and investigated as an example median endemic country.

## Methods

### Cohort definition

A population cohort of CHB patients was constructed from a recent meta-analysis of age- and region-specific hepatitis B surface antigen (HBsAg) prevalence in Turkey [6]. We projected these age-stratified HBsAg prevalence from the meta-analysis to the total age-specific Turkish population numbers, which was a total of 71.5 million in 2009 [7].

The HBsAg positive cohort was first divided into two groups, active and inactive CHB, based on hepatitis B e-antigen status, HBV DNA level, and serum alanine aminotransferase (ALT) level. The age-specific distributions of these factors were derived from a newly constructed patient database of the gastroenterology departments of the University of Ankara, and a state hospital in Ankara (Turkiye Yuksek Ihtisas Hastanesi) with 1,453 newly diagnosed CHB patients. Both of these hospital departments receive patients from around the country, which supposedly means that the constructed patient data is heterogeneous. In Turkey, almost all CHB cases are detected at the hospital, of which some patients are coincidentally detected during other medical procedures. The differentiation of active and inactive CHB is essential since progression of the disease is different in these two groups. Patients with high HBV DNA levels HBV DNA ≥10^4^ copies/mL and elevated ALT (>2 × ULN) have potentially progressive liver disease and are candidates for HBV antiviral therapy [2], while those with low or undetectable HBV DNA and normal ALT levels usually are inactive HBsAg carriers with a low risk of disease progression. Lastly, we classified the active CHB patients into four categories, namely HBeAg (+) and HBeAg (−) CHB with or without cirrhosis, respectively, using age group-specific proportions from large HBeAg-positive and HBeAg-negative clinical trials [8, 9].

### Model and clinical probability estimates

We evaluated the cohort of treatment-naïve active CHB patients for mortality, morbidity, impact of treatment and cost-effectiveness of various treatment strategies for a follow-up time of 20 years, thus the cycle length was 21, and the half cycle correction was applied with the TreeAge Pro 2009 software (TreeAge Software, Inc., MA, USA). The model uses annual probabilities of transition from CHB to virologic response, and of progression to cirrhosis, decompensated liver disease or hepatocellular carcinoma, liver transplantation, and finally death. The natural history and treatment related annual probabilities are obtained mostly from systematic reviews (Tables 1, 2) [10–36]. Other causes of death not related to liver disease are included in the model, as age-specific mortality rates derived from the Turkish statistics institute [7]. The probabilities of receiving a liver transplant were calculated based on personal communications with six major liver transplantation centres distributed throughout Turkey. We received reports that included annual numbers of liver transplants due to HBV related decompensated cirrhosis and HCC. From these figures, we calculated that there are annually around 500 liver transplantations performed in Turkey, of which about 150 are for HBV alone (no co-infections included). Out of these 150 liver transplantations, 120 are due to decompensated cirrhosis, and 30 to HCC. This corresponds to an annual probability of receiving a liver transplant for decompensated cirrhosis of 24 and 6 % for HCC.

**Table 1:** Annual transition estimates of the natural history of chronic hepatitis B by initial state

Initial state	Outcome	Estimate (%)*	References^a^
Chronic hepatitis B e+	Resolution	6.9 (2.0–23)	[10]
	Cirrhosis	3.8 (1.6–5.9)	[11]
	Hepatocellular carcinoma	0.3 (0.3–0.6)	[11]
	Chronic hepatitis B e−	1.9 (1.0–3.8)	[11]
Chronic hepatitis B e−	Resolution	1.6 (0.0–11)	[10]
	Cirrhosis	9.7 (2.9–16.3)	[11]
	Hepatocellular carcinoma	0.3 (0.3–0.6)	[11]
Cirrhosis e+	Decompensated cirrhosis	3.9 (2.0–7.9)	[12–14]
	Hepatocellular cancer	1.8 (0.9–3.8)	[12–14]
	HBV related death	3.1 (3.1–3.8)	[12–14]
Cirrhosis e−	Decompensated cirrhosis	2.7 (1.4–5.4)	[12–14]
	Hepatocellular cancer	2.9 (1.0–5.6)	[12–14]
	HBV related death	3.1 (3.1–3.8)	[12–14]
Decompensated Cirrhosis	Liver transplantation	23 (15–25)	Personal communication^b^
	HBV related death	26 (15–62)	[12–14]
Hepatocellular carcinoma	Liver transplantation	6 (3.0–7.0)	Personal communication^b^
	HBV related death	35 (20–60)	[10]
Liver transplant	HBV related death	6.6 (2.0–12)	[10]

*HBV* hepatitis B virus

* Ranges are shown in parentheses

^a^Estimates derived from European cohort studies

^b^The probabilities of receiving a liver transplantation for decompensated cirrhosis and hepatocellular carcinoma were calculated on the basis of data from six major transplant centers in Turkey

**Table 2:** Treatment-related annual transition estimates

Initial state	Outcome	Estimate (%)									
		Lamivudine		Entecavir^h^		Adefovir salvage		Tenofovir^i^		Tenofovir salvage^j^	
HBeAg status		**+**	**−**	**+**	**−**	**+**	**−**	**+**	**−**	**+**	**−**
CHB initial therapy^a^	Sustained virological response	20	10	22^b^	11^b^	12	10	23	11	19	11
	Cirrhosis^c^	0.5	1.2	0.2	0.6	0.5	1.2	0.2	0.6	0.5	1.2
	Hepatocellular carcinoma^f^	0.2	0.2	0.2	0.2	0.2	0.2	0.2	0.2	0.2	0.2
CHB long-term therapy	Sustained virological response	24	10	27^b^	11^b^	12	10	27	11	19	11
	Cirrhosis^c^	0.5	1.2	0.2	0.6	0.5	1.2	0.2	0.6	0.5	1.2
	Resistance: year 1	23^d^	23e	0.1	0.1	6^e^	6^e^	0	0	0	0
	Year 2	42^d^	42^d^	0.3	0.3	21^e^	21^e^	0	0	1	1
	Year 3	53^d^	53^d^	0.4	0.4	21^e^	21^e^	0.4	0.4	1	1
	Year 4	70^d^	70^d^	0.8	0.8	21^e^	21^e^	0.8	0.8	1	1
	Year 5	74^d^	74^d^	1	1	21^e^	21^e^	1	1	1	1
	Hepatocellular carcinoma^f^	0.2	0.2	0.2	0.2	0.2	0.2	0.2	0.2	0.2	0.2
Resistant CHB long-term therapy	Sustained virological response	4.5	0	5^b^	0.5^b^	4.5	0	5	0.5	5	0.5
	Cirrhosis^c^	2.7	6.2	2.7	6.2	2.7	6.2	2.7	6.2	2.7	6.2
	Hepatocellular carcinoma^f^	0.4	0.4	0.4	0.4	0.4	0.4	0.4	0.4	0.4	0.4
Cirrhosis initial therapy	Sustained virological response	20	10	22^b^	11^b^	12	10	23	12	19	11
	Hepatocellular carcinoma^f^	0.9	1.5	0.9	1.5	0.9	1.5	0.9	1.5	0.9	1.5
Cirrhosis long-term therapy	Sustained virological response	24	10	27^b^	11^b^	12	10	27	11	19	11
	Resistance: year 1	23^d^	23^d^	0.1	0.1	6′′	6′′	0	0	0	0
	Year 2	42^d^	42^d^	0.3	0.3	21^e^	21^e^	0	0	1	1
	Year 3	53^d^	53^d^	0.4	0.4	21^e^	21^e^	0.4	0.4	1	1
	Year 4	70^d^	70^d^	0.8	0.8	21^e^	21^e^	0.8	0.8	1	1
	Year 5	74^d^	74^d^	1	1	21^e^	21^e^	1	1	1	1
	Decompensated cirrhosis	1.9	1.9	1.9	1.9	1.9	1.9	1.9	1.9	1.9	1.9
	Hepatocellular carcinoma	1.6	1.6	1.6	1.6	1.6	1.6	1.6	1.6	1.6	1.6
	Death HBV	2.4	2.4	2.4	2.4	2.4	2.4	2.4	2.4	2.4	2.4
Resistant cirrhosis long-term therapy	Sustained virological response	4.5	0	5^b^	0.5^b^	4.5	0	5	0.5	5	0.5
	Decompensated Cirrhosis	7.9	7.9	7.9	7.9	7.9	7.9	7.9	7.9	7.9	7.9
	Hepatocellular carcinoma	1.8	2.9	1.8	2.9	1.8	2.9	1.8	2.9	1.8	2.9
	Death HBV	3.1	3.1	3.1	3.1	3.1	3.1	3.1	3.1	3.1	3.1
Decompensated Cirrhosis	Liver transplantation^g^	3.3	3.3	3.3	3.3	3.3	3.3	3.3	3.3	3.3	3.3
	Death HBV	26	26	26	26	26	26	26	26	26	26
Hepatocellular carcinoma	Liver transplantation^g^	1.2	1.2	1.2	1.2	1.2	1.2	1.2	1.2	1.2	1.2
	Death HBV	35	35	35	35	35	35	35	35	35	35
Liver transplantation	Death HBV	6.6	6.6	6.6	6.6	6.6	6.6	6.6	6.6	6.6	6.6

Estimates from Kanwal et al. [10, 15]

^a^Initial therapy is 12 months (48 weeks) of therapy

^b^Estimates from recent clinical trials: Chang et al. [16], Lai et al. [17] and Colonno et al. [18]

^c^Estimates calculated by the author, based on the assumption that the natural progression rates of chronic hepatitis B are reduced by antiviral therapy. Estimates derived from natural history estimate similar to Kanwal’s assumption of no progression of disease in HBeAg seroconversion, we assume no progression of disease in case HBV DNA is undetectable by PCR. In the papers from Chang and Lai full suppression of HBV DNA was observed in 80 % with a high resistance profile drug, and 90 % with a low resistance profile drug. We took these percentages for our calculations. Refs. [16, 17]

^d^Estimates for Lamivudine resistance from Lai et al. [19] and Moskovitz et al. [20]

^e^Adefovir salvage resistance estimates from Lee et al. [21], Chen et al. [22] and Yeon et al. [23]

^f^Estimates based on reduction of progression rates by nucleoside analogue therapy of 50 % Ref. [24]

^g^The probabilities of receiving a liver transplantation for decompensated cirrhosis and hepatocellular carcinoma were calculated on the basis of data from six major transplant centres in Turkey

^h^Estimates for entecavir resistance from Colonno et al. [18, 25] and Tenney et al. [26]

^i^Tenofovir monotherapy estimates Ref. [27]

^j^Tenofovir salvage scenario estimates from van Bommel et al. [28], Sarin et al. [29], van Bommel et al. [30] and Reijnders et al. [31]

### Scenario analysis

The following treatment options used by clinicians in Turkey were analyzed:

*Natural History* (*no antiviral treatment*) scenario: In this scenario, which is the base case scenario, active CHB patients progress according to the natural history, following annual rates of progression derived from systematic reviews (Table 1). Since the disease progression rates differ among European and Asian cohort studies [11], we only implemented in the model the annual progression rates derived from European cohort studies. We assumed that patients were followed clinically but did not receive antiviral therapy for CHB. Patients followed the natural history according to their HBeAg and disease status (with or without cirrhosis). Resolution was defined as seroconversion to anti-HBe in HBeAg positive patients, and as persistent HBV DNA suppression and ALT normalization in HBeAg negative patients. We assumed that all patients received regular ongoing care once complications occur.

*Lamivudine monotherapy* scenario: In this scenario, patients received 100 mg orally once daily with the first licensed antiviral HBV drug that is associated with a high incidence of resistance [24]. Such monotherapy is still being practiced in many countries with limited resources [5, 37]. We defined sustained virological response (SVR) in HBeAg positive patients as HBe-antigen loss and development of antibodies against HBeAg (anti-HBe).

*Entecavir monotherapy* scenario: Patients in this strategy received 0.5 mg entacavir once daily [38, 39]. The treatment related probability estimates are shown in Table 2.

*Tenofovir monotherapy* scenario: In this scenario patients received 300 mg of tenofovir for a continuum of 20 years. The annual probability of resistance in this scenario was 0 % for the first and second years of treatment.

*Adefovir salvage* scenario: In this scenario, patients initially receive lamivudine. Once resistance occurs, patients are salvaged add-on by add-on adefovir. Patients without resistance continued to receive lamivudine.

*Tenofovir salvage* scenario: In this more up-to-date scenario, patients who have developed resistance during lamivudine therapy are switched to treatment with tenofovir [31].

*Pegylated Interferon, followed by tenofovir* scenario: In this scenario patients receive 180 mcg/mL of pegylated interferon once a week subcutaneously, for 48 weeks. If the patients do not respond or relapse, they start tenofovir in the following year. The annual transition rates for SVR after 72 weeks of Peg-IFN was 30 % for HBeAg-positive and 20 % for HBeAg-negative patients [32–34, 40]. The withdrawal rate was 2 and 5 % for HBeAg-positive and negative patients, respectively [35].

*Roadmap concept* scenario: In this scenario we applied the ‘roadmap concept’ [41] to the sub-group of CHB non-cirrhotic HBeAg-negative cases treated with lamivudine, due to its low price, which continues to be widely used in HBV endemic areas. Patients with HBV DNA levels <10^7 ^copies/mL start therapy with lamivudine; after 24 weeks virologic response on treatment is assessed. If HBV DNA is undetectable (50 UI/mL, 300 copies/mL), patients continue their treatment with lamivudine until resistance or virologic breakthrough occurs, after which patients are switched to tenofovir. However, if HBV DNA is above 300 copies/mL at 24 weeks, lamivudine is switched to tenefovir monotherapy already at week 24. 71 % of patients are expected to become HBV DNA negative at week 24 of treatment [42]. Annual resistance rate in these patients on lamivudine treatment is 2 % [43, 44]. Annual rate of HBV DNA relapse is 8.2 % (28 % at 4 years) [44].

### Model assumptions

An assumption was that HBeAg-positive non-cirrhotic patients stop treatment after receiving one year consolidation treatment after HBeAg seroconversion and achieving undetectable HBV DNA levels [2], while HBeAg-negative patients continue treatment [38] for the follow-up period of 20 years. Also, our model assumes that the resistance for entecavir and tenofovir scenarios stays low as recent studies report. After the third year of treatment tenofovir resistance is assumed to be the same as entecavir. We took different time points to assess the outcomes for pegylated interferon and nucleos(t)ide analogues. For the Peg-IFN scenario we assumed that the non-responders continued with long-term tenofovir treatment both in HBeAg-positive and negative patients. For the road map concept the annual resistance of 2 % estimate was derived from the GLOBE telbivudine versus lamivudine trial [43].

### Cost and utility estimates

Medical costs are obtained from a retrospective analysis of the medical records of a sample of 542 patients (3,000 hospital admissions), where a random sample of patients was selected from inactive carriers, CHB active, cirrhosis, HCC and liver transplantation cases. An average annual medical treatment cost (excluding antiviral treatment) per patient in each health state was calculated (unpublished work). Data extracted for outpatient visits included information of visits, diagnosis, type of examination, signs and symptoms, laboratory tests, and procedures. Outpatients costs were mainly costs of laboratory tests, examination and consult. Information for inpatients included the length of stay, bed costs, surgical procedures, radiation treatment and chemotherapy. Annual cost was calculated as: cost per visit × visit per year + costs per admission × admission per year. The costs of antiviral drugs are obtained from the Turkish Ministry of Health [45]. A wide range of age-specific health state utilities are obtained from a multinational study on chronic hepatitis B [46]. Table 3 contains the specific cost and utility estimates. All costs and utilities were discounted at a rate of 3 % per year [47].

**Table 3:** Annual costs and health state utilities for chronic hepatitis B

Parameter	Base-case estimate TL (€)	(range)	References
Drug costs (year 2010 values)			
Lamivudine treatment (100 mg)	1,176 (585)	884–1,470 (439–731)	[45]
Adefovir salvage treatment (10 mg)	12,012 (5,976)	9,009–15,015 (4,482–7,470)	[45]
Entecavir treatment (0.5 mg)	11,292 (5,618)	8,469–14,115 (4,214–7,022)	[45]
Tenofovir (300 mg)	8,028 (3,994)	6,021–10,035 (2,996–4,992)	[45]
Peg-INF alfa 2a (INJVL 180MCG/ML)	19,344 (9,624)	14,508–24,180 (7,218–12,030)	[45]
Medical management costs			Personal communication^a^
Monitoring of CHB	720 (358)	540–900 (269–627)	
Compensated Cirrhosis	1,204 (602)	903–1,505 (452–752)	
Decompensated Cirrhosis	5,364 (2,668)	4,023–6,705 (2,001–3,335)	
Hepatocellular carcinoma	14,300 (7,114)	10,725–17,875 (5,336–8,892)	
Liver transplantation	174,050 (86,592)	130,538–217,562 (64,944–108,240)	
Health state utilities^b^			
Durable response to treatment	1.00	(0.95–1.00)	[46]
Chronic HBV	0.68	(0.66–0.70)	[46]
Compensated cirrhosis	0.69	(0.66–0.71)	[46]
Decompensated cirrhosis	0.35	(0.32–0.37)	[46]
Hepatocellulr carcinoma	0.38	(0.36–0.41)	[46]
Liver transplantation	0.67	(0.64–0.69)	[46]

*CHB* chronic hepatitis B, *HBV* hepatitis B virus

^a^Obtained from a retrospective analysis of medical records of a sample of 3,000 hospital admissions unpublished work

^b^See Levy et al. (Ref. [46]) for the age-specific utilities

### Outcomes

By applying the Markov cohort analysis, the cumulative mortality, and the cumulative probability of developing cirrhosis, decompensated cirrhosis, HCC, and getting a liver transplant were quantified for a 20-year time period. We measured costs (2010 Euro and Turkish Lira), quality adjusted life years (QALYs), and the incremental cost-effectiveness ratio (ICER), to determine the additional cost to obtain one QALY. The guidelines of economic submission to the BMJ was used for this cost-effectiveness analysis [48].

### Sensitivity analysis

To study the effect of uncertainty of the robustness of our results, we performed a sensitivity analysis on the low and high ranges of the transition estimates in the natural history scenario (Table 1). First, a so called best case scenario was assessed by applying the high range of achieving spontaneous virological response, and the low ranges for the estimates of disease progression. Second, a worst case scenario was assessed by applying the low rates for spontaneous virological response, and the high ranges for the disease progression estimates.

We assume that after seroconversion occurs, patients are allowed a 6 month therapy, and NAs are discontinued. A recent publication suggests continuation of long-term nucleos(t)ide analogue treatment, irrespective of the occurrence of HBeAg seroconversion in HBeAg-positive patients. Following this recent finding, an alternative scenario was assessed where treatment was continued in HBeAg-positive patients.

In addition, we performed a Monte Carlo simulation assuming that all variables followed a triangular distribution, due to its continues distribution, with base case, low and high range of values. We simulated 10,000 trials and plotted the results on cost-effectiveness acceptability curve stratified by cost-effectiveness thresholds to determine which treatment to use under different budgetary restraints.

## Results

### Cohort

Table 4 shows the total population of Turkey in 2009 with the age-specific prevalence of HBsAg. Around 3.2 million people (4.6 % of the total population) were estimated to be HBsAg carriers, with 22.6 % of them having HBeAg-positive CHB and 77.4 % having HBeAg-negative CHB. The total number of patients with active CHB was about 828,000 or 25 % of the total HBsAg-positive cohort, of which 57 % had HBeAg-positive and the rest HBeAg-negative CHB. The proportion that had cirrhosis in the active CHB cohort was 13 %.

**Table 4:** Age group specific distribution of chronic hepatitis B in Turkey by HBeAg and stage of liver disease

Age group					Active CHB		Cirrhosis		Chronic hepatitis (no cirrhosis)	
(years)	Population	HBsAg+ (%)	HBeAg+	HBeAg−	HBeAg+	HBeAg−	HBeAg+ (%)	HBeAg− (%)	HBeAg+	HBeAg−
0–14	18,788,587	533,596 (2.84)	283,828	249,768	90,621	47,586	1,818 (2)	2,379 (5)	88,808	45,207
15–24	12,441,662	490,201 (3.94)	176,473	313,729	91,504	45,804	1,830 (2)	2,290 (5)	89,674	43,514
25–34	12,328,944	784,121 (6.36)	159,961	624,160	113,892	132,322	6,834 (6)	9,263 (7)	107,058	123,059
35–44	10,070,734	624,386 (6.20)	62,439	561,947	35,222	96,610	2,466 (7)	14,491 (15)	32,756	82,118
45–54	7,927,348	437,590 (5.52)	22,655	414,935	11,923	74,353	2,981 (25)	20,819 (28)	8,943	53,534
55–64	5,066,402	184,924 (3.65)	12,753	172,170	5,070	41,595	1,673 (33)	21,214 (51)	3,397	20,382
65+	4,893,423	197,205 (4.03)	18,260	178,945	4,565	37,280	0	20,877 (56)	4,565	16,403
Total	71,517,100	3,252,022 (4.57)	736,367	2,515,655	352,797	475,550	17,595 (9)	91,333 (19)	335,202	384,217

*CHB* chronic hepatitis B, *HBeAg* hepatitis B e antigen, *HBsAg* hepatitis B surface antigen

### Mortality and morbidity in the active CHB cohort

The estimated age-specific CHB burden in a 20-year follow up is shown in Table 5 for the natural history scenario. If the cohort of 828,347 individuals remains untreated, it is estimated that 256,788 (31 %) will die due to liver related complications. Within a 20-year period, 11 % will have developed decompensated cirrhosis, 12 % HCC and 6 % will need liver transplantation. At the entry into the cohort in the year 2009, 108,928 (13 %) cases were estimated to be already in the cirrhotic stage. By the year 2029, another 247,261 (30 %) cases will have developed cirrhosis if left untreated, and this will have led to a cumulative number of 356,189 (43 %) patients with cirrhosis in the eligible cohort.

**Table 5:** Age-specific clinical outcome of active chronic hepatitis B by HBeAg status in the natural history scenario

HBeAg status	*n*	Outcome				
Age-group (years)		Cirrhosis (%)	Decompensated Cirrhosis (%)	HCC (%)	Liver transplant (%)	Death (%)
HBeAg+						
<15	90,621	819 (1)	863 (1)	1,234 (1)	503 (0,5)	2,574 (3)
15–24	91,504	833 (1)	877 (1)	1,257 (1)	512 (0,5)	2,617 (3)
25–34	113,892	35,401 (31)	12,082 (11)	8,461 (7)	5,776 (5)	27,276 (24)
35–44	35,222	10,966 (31)	3,694 (11)	2,561 (7)	1,769 (5)	8,316 (24)
45–54	11,923	3,783 (32)	1,741 (15)	1,050 (9)	825 (7)	3,842 (32)
55–64	5,070	1,630 (32)	779 (16)	452 (9)	359 (7)	1,689 (33)
65+	4,565	1,574 (34)	241 (5)	206 (5)	109 (2)	526 (12)
All HBeAg+	352,797	55,006 (16)	20,277 (6)	15,221 (4)	9,853 (3)	46,840 (13)
HBeAg−						
<15	47,586	424 (1)	680 (1)	1,153 (2)	412 (1)	2,465 (5)
15–24	45,804	406 (1)	657 (1)	1,110 (2)	400 (1)	2,389 (5)
25–34	132,322	99,242 (75)	22,392 (17)	26,464 (20)	11,770 (9)	67,484 (51)
35–44	96,610	73,924 (76)	16,424 (17)	19,322 (20)	8,695 (9)	50,237 (52)
45–54	74,353	54,278 (73)	12,640 (17)	15,614 (21)	6,692 (9)	39,407 (53)
55–64	41,595	28,285 (68)	7,071 (17)	8,319 (20)	3,744 (9)	22,045 (53)
65+	37,280	25,723 (69)	4,846 (13)	5,592 (15)	2,237 (6)	13,794 (37)
All HBeAg−	475,550	323,374 (68)	76,088 (16)	90,355 (19)	38,044 (8)	223,509 (47)
Total	828,347	356,189 (43)	91,118 (11)	99,402 (12)	49,701 (6)	256,788 (31)

### Impact of antiviral treatment on burden of disease

Treating the cohort with lamivudine monotherapy will decrease the mortality from 256,787 (31 %) to 124,253 (15 %) of cases, and when salvage therapy without delay is applied once patients become resistant to lamivudine, mortality will further decrease to 49,700 (6 %) cases. With the Peg-IFN (followed by tenofovir) strategy mortality will be reduced to 82,834 (10 %) cases. Treating the same patients with entecavir or tenefovir monotherapy will decrease the mortality to 41,417 liver related deaths (5 %).

### Cost-effectiveness

**Fig. 1:**
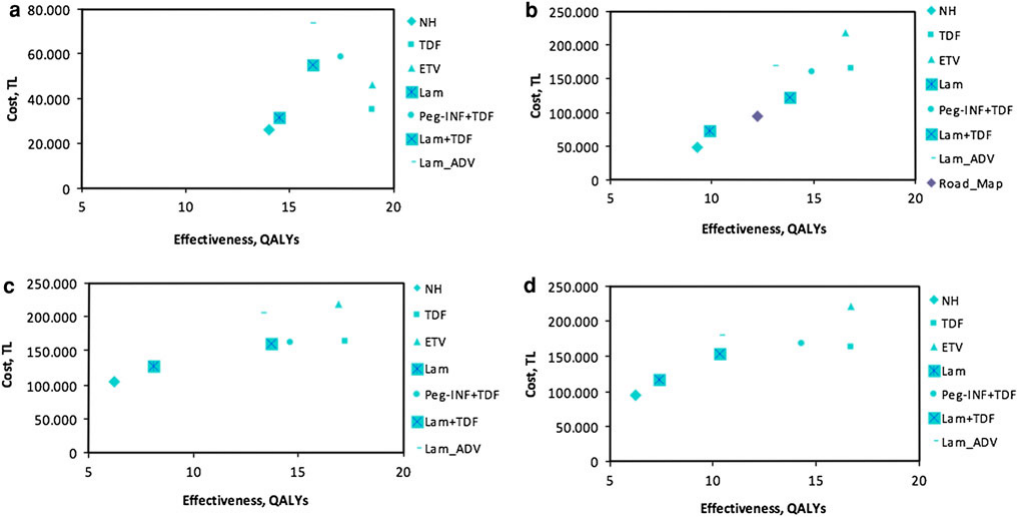
Results of cost-effectiveness analysis stratified by hepatitis B e antigen (HBeAg) and stage of liver disease: **a** HBeAg-positive (non-cirrhosis) **b** HBeAg-negative (non-cirrhosis) **c** HBeAg-positive (cirrhosis) **d** HBeAg-negative (cirrhosis). Results plotted on a cost-effectiveness plane. The *x*-axis represents the gain in QALYs with each strategy, and the *y*-axis the total healthcare costs (year 2010 values). *NH* natural history, *Lam* lamivudine, *Lam* *+* *ADV* adefovir salvage therapy, *Peg_IFN* *+* *TDF* pegylated interferon followed by tenofovir, *ETV* entecavir, *TDF* tenofovir

A plot of the outcomes of the various strategies on the cost-effectiveness plane according to HBeAg and disease status is shown in Fig. 1. The total costs, QALYs gained, incremental QALYs, incremental costs and ICERs for each scenario are presented in Table 6.

**Table 6:** Base case results of various scenarios: costs, quality adjusted life years (QALYs) gained, incremental QALYs, incremental costs and incremental cost-effectiveness ratios (ICERs)

Treatment	NH*		Lam		Lam + ADV		Lam + TDF	
HBeAg status	+	−	+	−	+	−	+	−
CHB (no cirrhosis)								
Cumulative costs (×1,000 TL (€))	25.7 (12.8)	48.2 (23.9)	31.6 (15.7)	72.6 (36.1)	73.4 (36.5)	168.5 (83.8)	54.7 (27.2)	122.5 (60.9)
Cumulative QALYs	14.0	9.3	14.5	9.9	17.0	13.0	17.5	13.8
Incremental costs (×1,000 TL (€))^a^	–	–	5.8 (2.9)	24.4 (12.1)	41.9 (20.8)	120.3 (59.9)	23.2 (11.5)	74.4 (37.0)
Incremental QALYs^b^	–	–	0.5	0.6	3.0	3.7	3.5	4.5
ICER (×1,000 TL (€)/QALY)	–	–	11.2 (5.5)	38.3 (19.0)	13.9 (6.9)	32.1 (16.0)	6.6 (3.2)	16.3 (8.1)
Cirrhosis								
Cumulative costs (×1,000 TL (€))	104.9 (52.2)	93.9 (46.7)	128.1 (63.7)	117.2 (20.1)	205.5 (102.2)	180.0 (89.6)	160.6 (79.9)	154.6 (76.9)
Cumulative QALYs	6.2	6.2	8.1	7.4	13.2	12.9	13.7	13.5
Incremental costs (×1,000 TL (€))^a^	–	–	23.3 (11.6)	23.2 (11.6)	100.7 (50.0)	86.1 (42.8)	55.8 (27.5)	60.6 (30.1)
Incremental QALYs^b^	–	–	1.9	1.2	7	6.7	7.5	7.3
ICER (×1,000 TL (€)/QALY)	–	–	12.5 (6.2)	20.1 (10.0)	14.5 (7.2)	12.9 (6.4)	7.5 (3.7)	8.3 (4.1)

Treatment	Peg INF + TDF		ETV		TDF		Roadmap
HBeAg status	+	−	+	−	+	−	−
CHB (no cirrhosis)							
Cumulative costs (×1,000 TL (€))	58.7 (29.2)	161.0 (80.1)	46.0 (22.9)	218.0 (108.5)	34.8 (17.3)	165.9 (82.5)	94.8 (47.1)
Cumulative QALYs	17.5	14.9	18.8	16.6	19.0	16.8	12.2
Incremental costs (×1,000 TL (€))^a^	27.1 (13.5)	112.9 (56.2)	14.4 (7.2)	169.9 (84.5)	3.2 (1.6)	117.7 (58.5)	46.6 (23.2)
Incremental QALYs^b^	3.5	5.6	4.8	7.3	5.0	7.6	2.9
ICER (×1,000 TL (€)/QALY)	7.8 (3.9)	20.0 (9.9)	3.0 (1.5)	23.2 (11.6)	0.6 (0.3)	15.6 (7.8)	15.6 (7.9)
Cirrhosis							
Cumulative costs (×1,000 TL (€))	162.9 (81.0)	168.2 (83.7)	218.5 (108.7)	220.8 (109.8)	163.5 (81.3)	163.1 (81.2)	–
Cumulative QALYs	14.6	14.3	16.9	16.5	17.2	16.7	–
Incremental costs (×1,000 TL (€))^a^	58.1 (28.9)	74.2 (36.9)	113.6 (56.5)	126.8 (63.1)	58.6 (29.2)	69.1 (34.4)	–
Incremental QALYs^b^	8.4	8.0	10.7	10.3	11.0	10.5	–
ICER (×1,000 TL (€)/QALY)	6.9 (3.4)	9.2 (4.6)	10.7 (5.3)	12.3 (6.1)	5.3 (2.6)	6.6 (3.3)	–

*NH* natural history, *Lam* lamivudine, *Lam* + *ADV* adefovir salvage therapy, *Peg_IFN* + *TDF* pegylated interferon followed by tenofovir, *ETV* entecavir, *TDF* tenofovir

* “NH (no treatment)” was the baseline strategy compared with other treatment strategies

^a^Difference in costs over NH

^b^Difference in healthy years over NH

#### Chronic hepatitis (non-cirrhosis)

The increasing health gain achieved over a period of 20 years for both HBeAg-positive and -negative patients has been assessed for lamivudine, the roadmap concept (for HBeAg-negative only), adefovir salvage, tenofovir salvage, pegylated interferon (followed by tenofovir), entecavir and tenofovir therapy strategies.

The natural history (no-treatment) strategy resulted in 14 and 9.3 QALYs and total discounted 20-year CHB related healthcare costs of 25,781 TL (€12,826) and 48,198 TL (€23,979) for the HBeAg-positive and negative cohort, respectively.

Both tenofovir and entecavir had equal incremental QALYs; however, entecavir compared to tenofovir, in a 20 year follow up period was 11,252 TL (€5,598) and 52,159 TL (€25,949) more expensive in HBeAg-positive and negative patients, respectively. The incremental cost-effectiveness ratio (ICER) of tenofovir versus no treatment was 638 TL (€318) and 15,573 TL (€7,747) for HBeAg-positive and negative patients, respectively.

#### CHB (cirrhosis)

The no-treatment strategy resulted in 6.2 QALYs and total healthcare costs of 104,859 TL (€52,168) and 93,954 TL (€46,743) for the cirrhotic HBeAg-positive and negative cohort, respectively. The lowest ICER was achieved with the tenofovir scenario versus no treatment which was 5,328 TL (€2,650) and 6,609 TL (€3,288) in the HBeAg-positive and negative cohort, respectively.

### Sensitivity analysis

The sensitivity analysis for the natural history scenario shows that, in comparison with the base case, in which the mortality of the active CHB cohort is 31 %, the mortality ranges from 17 % in the best case scenario to 42 % in the worst case scenario. When assessed by subgroups, in the worst case scenario, mortality ranges from 4 to 28 % for HBeAg-positive chronic hepatitis, from 8 to 35 % for HBeAg-negative chronic hepatitis, and from 62 to 91 % for cirrhosis independent of HBeAg status.

The ICER outcomes analysed when antiviral therapy were continued irrespective of HBeAg-seroconversion, varied according to the therapy chosen. Tenofovir and entecavir monotherapy had an ICER of 22,100 TL (€11,000) and 36,800 TL (€18,000), respectively. The ICER for lamivudine monotherapy, lamivudine/adefovir salvage and lamivudine/tenofovir salvage were, 56,200 TL (€27,900), 51,200 TL (€25,400) and 26,200 TL (€13,000), respectively. Pegylated interferon (followed by tenofovir) had an ICER of 27,600 TL (€13,700).

**Fig. 2:**
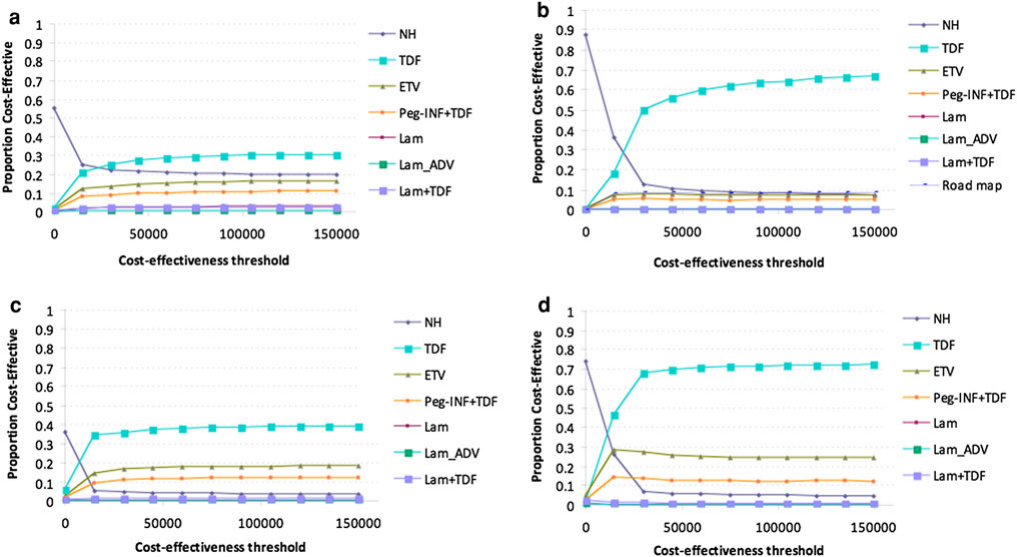
Cost-effectiveness acceptability curves showing the probabilities of net benefits achieved by each strategy for different willingness to pay thresholds (the maximum amount a person is willing to pay for a good) in HBeAg-positive (non-cirrhosis) (**a**), HBeAg-negative (non-cirrhosis) (**b**), HBeAg-positive (cirrhosis) (**c**), and HBeAg-negative (cirrhosis) (**d**). The vertical axes represent the probability of cost-effectiveness. The horizontal axes represent willingness-to-pay threshold to gain one additional quality adjusted life year (QALY). *NH* natural history, *Lam* lamivudine, *Lam* *+* *ADV* adefovir salvage therapy, *Peg_IFN* *+* *TDF* pegylated interferon followed by tenofovir, *ETV* entecavir, *TDF* tenofovir

The World Health Organization defines the threshold value for intervention cost-effectiveness as three times the gross national income (GNI) of a country. The threshold value for Turkey is 47,280 TL (€20,124) [49]. The probabilistic sensitivity analysis indicated that the no-treatment strategy was preferred at cost-effectiveness thresholds less than approximately 30,000 TL (€14,925) per QALY, and tenofovir had the highest probability of being optimal above this threshold (Fig. 2) for the HBeAg-positive (non-cirrhosis) patients. For the HBeAg-negative (non-cirrhosis) patients, tenofovir had the highest probability of being optimal above 30,000 TL (€14,925) per QALY.

For the HBeAg-positive cirrhotic patients, at a 15,000 TL (€7,462) per QALY threshold, tenofovir had the greatest net health benefit in 34 % of the simulations, and pegylated Interferon (followed by tenofovir) in 10 % of the simulations. In HBeAg-negative cirrhotic patients, tenofovir had a net health benefit of 46 % and pegylated interferon (followed by tenofovir) 14 % at a 15,000 TL (€7,462) per QALY threshold.

### Program costs for treating eligible patients

In addition to the cost and QALY gained per patient, we calculated the total program costs if the active CHB patients are identified and treated with the most cost-effective strategy. Tenofovir monotherapy had the lowest ICER for all sub-groups (HBeAg-positive; 638 TL (€318), HBeAg-negative; 15,573 TL (€7,747), HBeAg-positive cirrhosis; 5,328 TL, (€2,650), HBeAg-negative cirrhosis; 6,609 TL, (€3,288)), with ICERs far below the 36,212 TL (€18,016) threshold value. If the total 828,347 active CHB patients (Table 5) are treated, it will cost about 4.6 billion TL (€2.3 billion) annually, if not treated the total costs are tripled due to progression to liver failure and the high costs of medical treatment (hospitalization) and the need for liver transplantation.

## Discussion

In a country where the estimated number of HBsAg-positive cases is more than 3.2 million, the total amount of treatment eligible patients, which was quantified through population data and the large patient database constructed for this study, is 828,000, and of these, around 108,000 are patients with liver cirrhosis. If these eligible patients are not identified and treated, about 12,800 deaths are expected to occur each year due to liver related complications, leading to a cumulative number of 256,788 (31 %) in 20 years. The number of liver transplant patients in Turkey is 400–500 per year and this treatment is covered by the health insurance [50]. If we would modestly assume that 50 % of liver transplantations are due to HBV, there will be a total of about 4,000 liver transplantations that will take place in 20 years, while the demand will be around 49,000, according to our estimates. On top of all the life years lost, and more severe treatment options such as liver transplantation are needed, the 20-year cumulative medical management cost of an untreated active HBeAg-positive and HBeAg-negative CHB (no-cirrhosis) patient will be 25,781 TL (€12,800), and 48,198 TL (€23,900), respectively.

If the estimated active CHB cohort is identified and treated with the most cost-effective drug, liver related mortality and morbidity can be reduced by almost 80 %. Comparing treatment scenarios to the no antiviral treatment scenario in all the sub-cohorts, the tenofovir strategy was the most cost-effective. The ICER for HBeAg-positive and negative CHB (non-cirrhosis), and HBeAg-positive and negative cirrhosis was 638 TL (€306), 15,573 TL (€7,800), 5,300 TL (€2,600), and 6,609 TL (€3,300), respectively. Both entecavir and tenofovir, compared to the do nothing scenario, had the same amount of health gain. A recent systematic review and Bayesian meta-analysis concludes that in the first year of treatment for CHB, tenofovir and entecavir are the most potent oral antiviral agents for HBeAg-positive patients, while for HBeAg-negative patients tenofovir is most effective [51]. According to net sold medication counts per year in Turkey, it was calculated that no more than 10 % of active CHB patients receive antiviral treatment [52], indicating a massive shortcoming in providing eligible patients with life prolonged and even life saving treatments. At individual level the association of disease progression with increased cost of disease management suggests that measures to prevent or delay progression of CHB related liver diseases will be economically beneficial. At population level, however, the impact of therapy on the overall number of people with chronic infection will remain limited as long as the majority of infected patients will not receive treatment due to lack of recourse for optimal treatment.

The future public health burden of chronic hepatitis B could potentially be reduced by antiviral treatment [53]. The recommendations by the Turkish Association for the Study of the Liver (TASL) [52] to treat eligible patients are in line with the European Association for the Study of the Liver [2] criteria, except that liver biopsy evidence is always required to start treatment in patients with no established cirrhosis. Almost all patients are reimbursed for treatment of viral hepatitis through the national insurance system in Turkey. A new modification issued in 2009 by the department within Turkish Health Authorities responsible for reimbursement decisions, states that lamivudine should be the first line therapy in all patients with viral load lower than 10^7 ^copies/mL. This is largely due to the low costs of lamivudine and to the recent data about on-treatment monitoring approach, using serum HBV DNA level as a predictor for efficacy and drug resistance. We assessed whether this scenario (roadmap concept) was cost-effective in an HBeAg-negative non-cirrhotic patients group, since sufficient data were available for this sub-group. The ICERs of both scenarios, roadmap concept [15,829 TL (€7,875)] and tenofovir monotherapy [15,573 TL (€7,747)], were equal. Although eight healthy life years were gained by tenofovir monotherapy while this was only three healthy life years gained for the roadmap concept scenario.

According to our outcomes, the roadmap concept could be an alternative strategy to consider for a country with a large HBeAg-negative disease, where tenofovir is not available. This scenario could also be suggested in resource poor settings, since the cumulative costs to treat are less compared to tenofovir monotherapy. Various studies have examined the cost-effectiveness of antiviral therapy for CHB and have concluded that treatment is cost-effective versus no treatment [10, 15, 54–56]. Kanwal et al. [10] found that lamivudine monotherapy strategy was more expensive and less effective than treatment with interferon or salvaged by adefovir. According to our analysis, lamivudine monotherapy was less effective as well, but was not more expensive compared to other treatment strategies. This can be explained by the fact that more than 5 years have elapsed between both studies during which the price of lamivudine has decreased. Buti et al. [57] concludes that first-line treatment with tenofovir is cost-effective for both HBeAg-positive and negative patients, in comparison to other antivirals. They also conclude that tenofovir was more effective than entecavir, which is in contrast to our results for which the efficacy equality was equal for both drugs.

A country with similar patient characteristics and health care system may benefit from our scenario analysis and outcomes related to the burden of disease. Considering the economic affordability in different countries, the cost-effectiveness thresholds may be different. It may be that in a country where the threshold is high, a more expensive but effective drug is cost-effective, while this might not be the case for this same drug in a country with a lower cost-effectiveness threshold. A review study by Barbieri et al. [58] on the generalizability of cost-effectiveness studies concludes that the differences in cost-effectiveness results between countries are not systematic, which makes inferences from one country to another difficult.

A limitation of our study is that we used simplified assumptions (e.g., we did not consider coinfection with other viruses or toxins such as alcohol that will accelerate progression), and we assumed the cohort to be static, so there were no new cases added to the cohort. Also, the assumption that the development of resistance both with entecavir and tenofovir for the coming 20 years will stay at 0–1 % per year may underestimate what will happen as longer term data are collected. We took a rather conservative approach by only including high HBV DNA and ALT >2 × ULN. If, like in the guidelines, we had taken elevated ALT levels but starting at 1 × ULN, the number of eligible patients would have increased. Another factor that surely plays an important role in the estimation of eligible patients is the inclusion of data from tertiary centres. In Turkey, data on viral hepatitis are collected at the provincial health directorate, but only for acute (incident) cases. Thus, the data on CHB patients is derived from clinical settings, of which not all patients coming to the hospital have active disease. Some patients are detected during the diagnostic process for other diseases and referred to the hepatology department. We conclude that the cohort data from Turkey are, therefore, likely to be biased towards more active CHB cases, which could mean that the number of eligible patients might be an overestimation. One way to account for this bias would be to implement new information systems and registries to facilitate the notification, counseling, and medical management of persons with CHB infections in countries with intermediate or high endemicity. Any attempt to predict the future is likely to be biased. Therefore, our projections and estimates regarding future treatment rates and liver-related deaths are only intended to provide a crude overview of the public health impact of antiviral therapy.

Identification of chronic hepatitis B infected individuals is essential to ensure that infected persons receive necessary care to prevent or delay onset of significant liver disease and services to prevent transmission to others. Achieving identification could be done by monitoring inactive cases annually, as is recommended in the guidelines. Antenatal screening should be routinely performs, and new information systems and registries should be implemented to facilitate the notification, counselling, and medical management of persons with chronic HBV infection in countries with intermediate and high endemicity. Given the substantial mortality and morbidity attributable to HBV related chronic liver diseases, the control of progression to cirrhosis, decompensated cirrhosis and liver cancer will continue to be an important public health priority. Third generation drugs, such as entecavir and tenofovir, with high effectiveness and low resistance profiles, should be made more affordable to help people with active chronic hepatitis B lead healthier lives.

## Electronic supplementary material

Below is the link to the electronic supplementary material.

Supplementary material 1 (DOC 32 kb)
