# Supplementary material for: The cost-effectiveness of treating chronic hepatitis B patients in a median endemic and middle income country
Source: Eur J Health Econ. 2012 Jul 20;13(5):663–76. doi: 10.1007/s10198-012-0413-8 (PMC3427846; doi:10.1007/s10198-012-0413-8)
Supplement: Supplementary file 1 — Supplementary material 1 (DOC 32 kb) [file 10198_2012_413_MOESM1_ESM.doc]

Markov state transition diagram. Patients either enter the model with chronic hepatitis B virus infection (CHB), or Cirrhosis. During each 1-year cycle, individual patients either remain in their assigned health state, or progress to a new health state.

Appendix for Model structure:

**CHB health state**: Patients enter the model with CHB. Over a 20 year period, these patients may develop compensated cirrhosis, develop hepatocellular cancer directly without progressing to cirrhosis, or remain with CHB until death. Patients achieving virologic response (either spontaneously or after treatment) do not develop cirrhosis and have a normal life expectancy.

In each 1-year Markov cycle, patients receiving therapy for HBV may either achieve a virologic response, develop viral resistance, or continue to receive long-term therapy.

**Cirrhosis health state**: Patients enter the model with compensated cirrhosis. Patients with compensated cirrhosis may develop decompensated cirrhosis (including ascites, variceal bleeding and encephalopathy). Hepatocellular cancer or death may develop at any stage of cirrhosis. Patients with decompansated cirrhosis and hepatocellular cancer are eligible for liver transplantation.

CHB

Cirrhosis

Resolution

HCC

Decompensated Cirrhosis

Liver transplant

Death Hepatitis

Mortality

Transition
